# Theoretical Food and Nutrient Composition of Whole-Food Plant-Based and Vegan Diets Compared to Current Dietary Recommendations

**DOI:** 10.3390/nu11030625

**Published:** 2019-03-14

**Authors:** Micaela C. Karlsen, Gail Rogers, Akari Miki, Alice H. Lichtenstein, Sara C. Folta, Christina D. Economos, Paul F. Jacques, Kara A. Livingston, Nicola M. McKeown

**Affiliations:** 1 Friedman School of Nutrition Science and Policy, Tufts University,150 Harrison Ave, Boston, MA 02111, USA; mkarlsen@une.edu (M.C.K.); gail.rogers@tufts.edu (G.R.); alice.lichtenstein@tufts.edu (A.H.L.); sara.folta@tufts.edu (S.C.F.); christina.economos@tufts.edu (C.D.E.); paul.jacques@tufts.edu (P.F.J.); 2 Nutritional Epidemiology Program at Jean Mayer USDA Human Nutrition Research Center on Aging at Tufts University, 711 Washington St, Boston, MA 02111, USA; akari.miki@tufts.edu (A.M.); kara.livingston@tufts.edu (K.A.L.); 3 Department of Medicine, Tufts University School of Medicine, 145 Harrison Ave, Boston, MA 02111, USA

**Keywords:** vegan, whole food plant-based, nutrient composition, Healthy Eating Index, HEI-2015, MyPlate

## Abstract

Public interest in popular diets is increasing, in particular whole-food plant-based (WFPB) and vegan diets. Whether these diets, as theoretically implemented, meet current food-based and nutrient-based recommendations has not been evaluated in detail. Self-identified WFPB and vegan diet followers in the Adhering to Dietary Approaches for Personal Taste (ADAPT) Feasibility Survey reported their most frequently used sources of information on nutrition and cooking. Thirty representative days of meal plans were created for each diet. Weighted mean food group and nutrient levels were calculated using the Nutrition Data System for Research (NDSR) and data were compared to DRIs and/or USDA Dietary Guidelines/MyPlate meal plan recommendations. The calculated HEI-2015 scores were 88 out of 100 for both WFPB and vegan meal plans. Because of similar nutrient composition, only WFPB results are presented. In comparison to MyPlate, WFPB meal plans provide more total vegetables (180%), green leafy vegetables (238%), legumes (460%), whole fruit (100%), whole grains (132%), and less refined grains (−74%). Fiber level exceeds the adequate intakes (AI) across all age groups. WFPB meal plans failed to meet the Recommended Dietary Allowances (RDA)s for vitamin B12 and D without supplementation, as well as the RDA for calcium for women aged 51–70. Individuals who adhere to WFBP meal plans would have higher overall dietary quality as defined by the HEI-2015 score as compared to typical US intakes with the exceptions of calcium for older women and vitamins B12 and D without supplementation. Future research should compare actual self-reported dietary intakes to theoretical targets.

## 1. Introduction

Apparent interest in plant-based diets continues to grow, as evidenced by the variety of cookbooks, blogs, and related websites [1,2,3,4,5,6] that have emerged in recent years. Whole food plant-based (WFPB) and vegan diets [7,8] similarly exclude all animal products, including red meat, poultry, fish, eggs, and dairy products. However, although also a vegan diet, a WFPB diet places a greater emphasis on minimizing or excluding all processed foods made with refined grains and added sugar, refined fats and oils, and salt [9]. Although many recipes and cookbooks written for vegans do emphasize fruits, vegetables, whole grains, and legumes, they also tend to be less rigid with respect to avoiding refined foods or ingredients [10]. A variety of professional organizations in the US [11,12,13] and elsewhere [14,15,16] have now issued dietary guidance statements on vegetarian or vegan diets. While only a small segment (~<4%) of the U.S. population identifies as following a specific, plant-based diet [7,8], the rising public interest in WFPB and vegan diets underscores the importance of assessing how their nutrient profiles, as promoted by their proponents through popularly available books and recipes, compare to US dietary recommendations. Such dietary recommendations include the Dietary Guidelines for Americans (DGA), which uses the concepts of adequacy (achieving recommended levels of nutrient intake) and moderation (staying within recommended limits of nutrient intake) to create MyPlate eating guidelines (https://www.choosemyplate.gov/).

Data from NHANES 2009–2010 suggests that Americans consume 60% of their calories from ultra-processed foods such as breads, cakes, cookies, pizza, French fries, salty and sweet snacks, and desserts, with only 5% and <1% of calories from fruits and vegetables, respectively [17]. WFPB and vegan diets generally promote practices consistent with higher dietary quality, such as an emphasis on greater intakes of whole grains and vegetables and avoidance of added sugars and refined foods [9]. Thus, there is growing interest in these diets as healthier alternatives to the standard American diet. However, certain concerns have been raised with respect to nutrient adequacy for diets that exclude all foods of animal origin. Without supplementation or the consumption of fortified foods such as plant milks and nutritional yeast, diets with no animal foods may provide inadequate levels of vitamins B12 and D [18,19,20]. Without emphasis on sea vegetables [21] and/or inclusion of iodized salt, they also may provide inadequate levels of iodine [13,22]. Knowledge about the nutrient composition of WFPB and vegan diets is important if clinicians are to engage with patients about the health benefits and concerns associated with these diets. Many recipes in published cookbooks and websites are available to the public who choose to adhere to WFPB or vegan diets [1,2,3,4,5,6], but how these promoted recipes and meal plans translate into usual nutrient intakes remains unknown. To date, most of the research on diet quality or nutrient content of vegan diets has focused on weight loss with nutrient intakes derived from only researcher-defined meal plans of three [23] or seven-day [18,24] and compared to DRIs. To our knowledge, the *theoretical* food and nutrient composition of diet-specific meal plans *as reported by self-identified followers* of WFPB or vegan diets has not been thoroughly assessed.

The objectives of this study were to estimate theoretical food and nutrient levels of WFPB and vegan diets using 30 days of meal plans derived from popular cookbooks and recipe websites as reported by self-identified diet followers and compare these food and nutrient levels to US dietary recommendations, including Dietary Reference Intakes (DRIs) and/or MyPlate meal recommendations.

## 2. Materials and Methods

### 2.1. Sources of Frequently Consumed Foods

Top sources of diet information were captured from the Adhering to Dietary Approaches for Personal Taste (ADAPT) Feasibility Survey [25]. In brief, this was a short, web-based survey designed to recruit self-identified popular diet followers. The survey was open for 8 weeks in 2015, from 14th July to 14th September, and a total of 13,787 participants consented to enroll. Participants identified the specific diet they followed by selecting a single diet from among multiple choice answers with a write-in option if their diet was not listed [25]. This survey included a free-text question that asked participants to report their sources of cooking and nutrition information (books and websites) for their respective diets. A total of 6372 responded to this optional question and listed at least one book source, and the two largest self-identified groups were selected for this analysis. Of the total responses to this question, 29% self-identified as WFPB followers (n = 1856) and 19% as vegan (n = 1218). We used data on sources of dietary information from a random sample of 200 participants’ responses from each of the WFPB and vegan diets.

### 2.2. Selection of Recipes and Meal Plans

Foods and nutrient levels in each of the diets were calculated in a multi-stage process. Survey responses to the question “What are your main sources of information on nutrition and cooking for the diet you currently eat?” were coded for mentions of unique sources of diet information (i.e., books or websites authored by different experts or organization). The majority of responses included two or more unique sources. The six most frequently cited diet information sources (top three books and top three websites) mentioned by at least 5% of the selected respondents were used to create representative meal plans. Books and websites from the same author were combined and considered to be the same source. A five-day meal plan from each of the six sources was created, for a total of 30 representative days, for both WFPB and vegan diet groups. 

Similarly, MyPlate plans posted online (*Sample Two-Week Menus and Sample Menus for a 2000 Calorie Food Pattern*) were used to create 21-day USDA compliant meal plans [26,27]. Meal plan data were collected and analyzed using Nutrition Data System for Research software version 2016 (NDSR 2016) developed by the Nutrition Coordinating Center (NCC), University of Minnesota, Minneapolis, MN. NDSR provides a complete nutrient profile for all foods in the database [28]. 

Ingredients consistent with recipe instructions were selected whenever possible. When recipe instructions were ambiguous, NDSR data-entry rules were used to select generic ingredient choices and standard portion sizes to maintain consistency. Food plans and recipes were generated and entered in full to NDSR (Micaela C. Karlsen) and subsequently divided by the number of servings to obtain single-serving portions. The accuracy of recipes and meal plans entered into NDSR was confirmed by a second reviewer (Akari Miki). Prior to comparison, all meal plan data were standardized to 2000 kcal/day.

Dietary supplements that were advised by at least two meal plan sources were entered as part of the WFBP or vegan meal plans using the 24-h supplement intake module in NDSR. Vitamin B12 (µg) was included in all six meal plan sources and five of the six sources included daily vitamin D (calciferol, µg) supplements. Specific doses of supplements that were specified by the meal plan sources were entered, however when doses were not provided a generic supplement was selected (~1000 µg of B12 and 25 µg of vitamin D based on typical available products). Both total (food plus supplements) and diet only nutrient levels were calculated.

### 2.3. Comparison of Mean Food and Nutrient Data

Food and nutrient data from the meal plans was generated by NDSR and analyzed using SAS 9.4 (SAS Institute, Cary, NC, USA). Mean intakes for selected nutrients and food groups were calculated for each diet. Top sources of information and cooking were identified and weighted based on the percent of mentions of that source (book or website) within each diet group. The percent for weighting each source was calculated as the number of mentions of the source out of the total number of mentions of the top six sources. Interestingly, the top six sources mentioned were the same among both WFPB and vegan diet followers; however, the percent of mentions for these sources differed slightly, leading to minor differences in weight between the two groups. This weighting approach was chosen because the nature of the question was open-ended, and most respondents cited multiple sources of information, so accounting for multiple data sources captures a more representative estimate of the overall diet guidance followed. Diet quality was assessed using the Healthy Eating Index 2015 (HEI-2015) [19], an index of overall dietary quality that measures adherence to the 2015 Dietary Guidelines for Americans [29], The scoring protocol for the HEI-2015 is presented in Table S1.

Since the meal plan sources were identical between the two diets, and the weighting scheme applied only differed slightly, marginal differences were observed in the mean composition of the vegan versus WFPB diets after applying the weighting. Therefore, to avoid redundancy of results, only WFPB meal plans results are presented in the main text, and vegan meal plan results are presented in Tables S2–S5. Mean nutrient and food group levels from meal plans were compared to mean nutrient levels from MyPlate. For those items in which the standard deviation is 50% of the point estimate or more, we examined the median difference with similar findings (data not shown). Nutrient content of the meal plans was compared to the relevant Dietary Reference Intakes (DRIs) from the Institute of Medicine [30] for male and female adults (ages 19–70 years) for the Nutrients of Concern as identified by the US Dietary Guidelines Advisory Committee [31]. The Nutrients of Public Health Concern [31] are those nutrients for which a majority of Americans have been found to have intakes below either the Estimated Average Requirement (EAR) or the Adequate Intake (AI), or above the Upper Limit (UL). To determine the theoretical nutritional quality of the meal plans, we applied the following criteria to estimated nutrient levels: (1) EAR and RDA for vitamin A (RAE, µg), vitamin D (calciferol, µg), vitamin E (mg), folate (µg), vitamin C (mg), calcium (mg), magnesium (mg), and iron (mg); (2) AI for potassium (mg) and fiber (g); (3) UL for sodium (g); and (4) recommendation from the 2015 US Dietary Guidelines for Americans [29] to limit saturated fat and added sugar to less than 10% of calories. Percent differences are expressed as the percent greater or lesser for the meal plans’ mean nutrient content as compared to the DRIs and MyPlate. These differences were calculated as % difference = (mean meal plan value/recommended value) * 100 − 100. 

## 3. Results

Food servings of WFPB diets compared to MyPlate are presented in Table 1 (Table S2 for vegan). Plant-based milks are grouped with dairy and contributed a small amount to the dairy food group.

**Figure 1:**
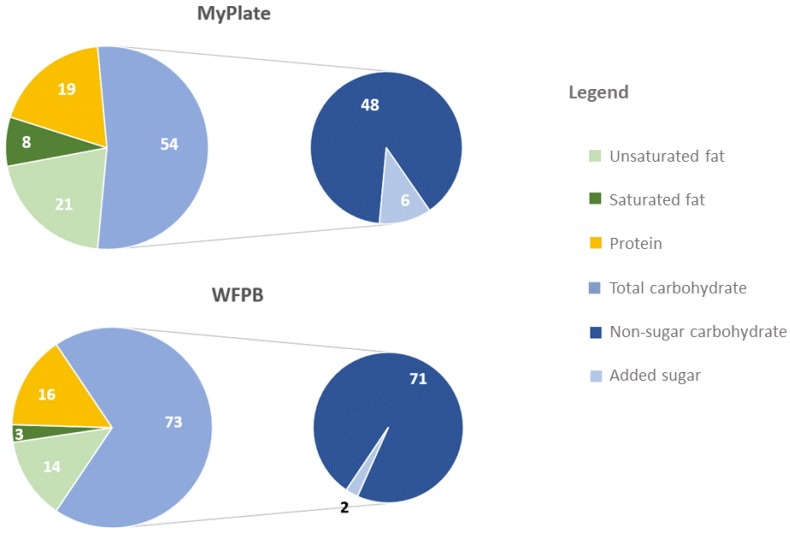
Macronutrient content (% of total energy) in MyPlate and whole-food plant-based WFPB meal plans.

Percent energy from macronutrients differed between the theoretical WFPB and MyPlate as shown in Figure 1. All protein in the WFPB diet is plant protein (16%), whereas 12% energy in the MyPlate meal plans was from animal protein and 7% from plant protein.

Calculated mean nutrient levels across meal plans are presented in Table 2 (Table S3 for vegan). The fiber content for MyPlate is 28 g/day and 70 g/day for WFPB. Percent energy from added sugar is estimated to be 6% for MyPlate and 2% for WFPB. Medians and interquartile ranges for food and nutrients are presented in Table S5 (both WFPB and vegan). 

Comparisons with recommended intake levels for the Nutrients of Public Health Concern [31] are presented in Table 3 (Table S4 for vegan). Estimated theoretical nutrient levels from the WFPB plans meet or exceed the RDAs for vitamin A, vitamin E, folate, vitamin C, magnesium, and iron for adult men and women. Estimated vitamin D levels are inadequate for vitamin D from food only (data not shown); however, when consuming supplements, the RDA is exceeded. EARs for calcium are met for men and women, and the RDA is met for men but not for women ages 51–70 years. Meal plans meet or exceed the AIs for potassium and fiber for men and women. Estimated sodium intake exceeds the UL for adult men and women. Levels of saturated fat and added sugar fall within the recommendation.

## 4. Discussion

This study uses a unique approach to create *theoretical* WFPB and vegan meal plans based on popular nutrition and cooking sources identified as part of a large online survey. This approach was novel in that previous work to analyze similar meal plans used sources selected by researchers, not individuals who self-identify as following a WFPB or vegan diet [18,23,24]. We observed that self-identified WFPB and vegan followers tended to report the same sources of nutrition and cooking information, and this translated into similar theoretical food and nutrient targets. While WFPB diets are a type of vegan diet, the distinction between the two may best be understood by investigating motivations for choosing one label or the other. In this survey, there was a higher prevalence of younger (18–35) diet followers among vegans compared to WFPB (26% versus 16%) as well as a lower prevalence of older adults (55+) (30% versus 42%) (unpublished data). Sixty-eight percent of WFPB versus 59% of vegans reported a past or current health diagnosis (unpublished data). These data may indicate some differences in motivation for following their diets due to health concerns. However, actual self-reported intakes among followers of these two diets were not examined, and, thus, diet quality in practice may differ between the two groups. Additionally, adherence to advice on supplement use may also differ in practice. Supplement advice derived from the meal plan sources appears to be aligned with products available in stores. In the case of B12, this means ingesting doses far larger than required for most individuals (~1000+ µg); however, due to the water-soluble nature of B12, toxicity due to excess is not a concern. Based on the results of this analysis, WFPB and vegan dietary guidance offer improved diet quality compared to typical US consumption patterns and current food-based and nutrient-based recommendations, with some important caveats. 

The diet quality for most US adults is poor with the mean HEI-2010 at 58 out of a possible 100 [32]. NHANES (2009–2010) data suggests that among US adults, approximately 14% of total energy comes from added sugars [33] while only 30% of calories comes from unprocessed or minimally processed foods such as meat or dairy, grains, legumes, and fruits and vegetables [17]. Furthermore, only 42% of Americans meet the Dietary Guidelines recommendation to limit added sugars to <10% of calories [34]. With respect to sodium intake, the average intake among US adults exceeds the UL by 48% (3412 mg/day [34] versus 2300 mg/day [30]), while average fiber intake is only 17 g/day [34], substantially lower than current Dietary Guidelines recommendations (14 g/day per 1000 kcal) [29]. Theoretically, if a person adhered to a WFPB or vegan diet, the quality of their diet would be superior to the typical American diet. 

The WFPB meal plans exceeded the MyPlate meal plan targets with respect to total vegetables, green vegetables, and nuts and seeds, as well as several nutrients including vitamin A, vitamin E, vitamin C, and folate. The lower HEI score of the WFPB diet is primarily due to the exclusion of dairy products. The contribution of energy from fat and carbohydrate differs substantially for WFPB relative to MyPlate, with WFPB displaying a “low-fat, high-carbohydrate” profile (17% fat, 73% carbohydrate). The intake of added sugar is substantially lower than the threshold of 10% to total energy [29] and lower than the American Heart Association threshold of 100 kcal/day for women and 150 kcal/day for men [35].

Higher intake of saturated fat has been associated with increased risk of cardiovascular disease [36], and randomized controlled trials have demonstrated that lowering consumption of saturated fat from approximately 15% to 6% of energy intake and replacing it with unsaturated fat significantly lowered low-density lipoprotein (LDL) cholesterol [37]. Among US adults, the average consumption of saturated fat intake is approximately 11% of total energy intake [34]. The theoretical saturated fat levels for WFPB diets fall within the more stringent guidelines of <6% set by the AHA for people with heart disease [37]. For most age and gender groups, fewer than 5% of Americans [38] surpass the AI for fiber, which is 14 g/1000 kcal/day (approximately 38 g/day for men and 25 g/day for women ages 19–50) [30]. Estimated dietary fiber content of MyPlate meal plans was close to the AI, while the WFPB meal plans surpassed the target at 70 g (84% more than the AI for daily fiber for men and 180% more for women). 

The micronutrient profile of the WFPB meal plans makes it relatively easy to achieve the EARs and RDAs for most nutrients; however, there are some important exceptions. Meeting the EAR or RDA for vitamin D on a WFPB diet may be challenging without supplementation, though vitamin D recommendations are based on the assumption of minimal sunlight exposure [30], which varies across individuals. From the derived WFPB meal plans, the EAR for vitamin B12 is achieved because of the inclusion of certain fortified foods in the menus such as plant milk and fortified nutritional yeast. In practice, however, without consuming fortified foods, WFPB followers would need to consume a supplement to achieve adequate B12 levels. These lower vitamin D and vitamin B12 intakes for the WFPB meal plans are consistent with an earlier study that analyzed seven single-day meal plans for a vegan diet and estimated that intake of vitamin B12 and vitamin D was 10% lower than the RDA [18]. Irrespective of diet, the Institute of Medicine recommends that all adults over age 50 consume a B12 supplement and/or fortified foods to compensate for reduced B12 absorption [30]. Consistent with this recommendation, the WFPB recipe sources identified in this analysis recommend daily vitamin D and B12 supplements. It is important to note again that it is unknown what actual dietary practices encompass; unpublished data from this same survey indicates that 83% of self-identified WFPB followers and 86% of self-identified vegan followers take supplements. Of these, 88% and 87% report taking single nutrient supplements, respectively. This is in contrast to national data from 2012 which indicates that 52% report using any supplements, and that 8.1% of adults report taking single B12 supplements while 19% report taking single vitamin D supplements [39]. For these particular nutrients, more research is needed to determine what actual intakes are in populations who report following nutrition advice that promotes use of specific supplements.

Calcium is an essential nutrient of particular importance for bone health, particularly among older Americans who are at greater risk for osteoporotic fractures [40]. The EARs for calcium are 800 mg/day for men 19–70 and women 19–50, and 1000 mg/day for men >70 and women >51 [30]. Nearly half of Americans do not meet the EAR for calcium [38]. In WFPB followers, exclusion of dairy products in these meal plans is the single largest contributor to the lower HEI-2015 scores. WFPB diets rely on non-dairy sources of calcium including tofu, green leafy vegetables, and fortified plant milks [41]. WFPB meal plans fail to meet the RDAs for calcium; however, they only fall short by −2 to −4% of the RDA for calcium among men. WFPB meal plans do meet the EAR for calcium for men and only fall 4% short of meeting the EAR for women. 

As individuals increasingly self-identify with following a WFPB or vegan diet, it is important for physicians with little nutrition training in medical school [42], as well as other healthcare professionals, to be equipped to discuss the strengths and drawbacks of such popularly promoted diet advice. Overall, WFPB diets are of higher quality compared to both MyPlate recommendations and actual US intakes estimated from NHANES; however, there are some important nutritional considerations. Individuals who exclude all foods of animal origin may be at risk of vitamin B12 and vitamin D deficiencies without taking supplements and are advised to consult with their physicians when making supplement decisions. 

The strength of this study was the characterization of food and nutrient levels through the use of recipes and meal plans that are popularly promoted by self-identified diet followers, as opposed to researcher-selected sources. Thus, these estimates may provide a more relevant window into the targeted practices of diet followers than officially published diet guidance [11,12,13,14,15,16]. Another strength of this meal plan analysis is that we collected sufficient numbers of meal plan data (30 days each) to achieve a more accurate estimate of nutrient levels. Other analyses of meal plans have used only three [23] to seven [18,24] days. Due to the high within-individual variation of dietary intakes, especially with respect to micronutrients, calculations based on three or seven days may not be sufficient to characterize true intake levels [43]. For example, the number of days required to estimate a person’s true intake has been calculated to be between 10–15 days for macronutrients and 3–24 days for most micronutrients [43]. 

A recognized limitation of this analysis centers on the methodology used to derived meal plans. Sources of nutrition and cooking information are limited to those in our self-selected sample, which is a group with internet access that is largely white and female, and who may or may not represent typical WFPB or vegan followers. In practice, the popular culture surrounding WFPB diets seems to emphasize health, while there are a broader variety of reasons reported for following a vegan diet that may or may not overlap with health concerns, such as ethical concerns about the treatment of animals [44]. It is possible that another sample of self-identified vegans recruited via organizations and thought leaders who emphasize non-health motivations might capture individuals with different motivations and who share fewer cooking and nutrition sources with our WFPB sample. As this survey did not capture actual dietary intake, it is unknown whether the choice of self-identified label as either WFPB or vegan in this sample speaks to differences in actual dietary intakes between the groups. 

While this analysis does not examine how actual dietary intake practices align with goals, knowledge of the targeted *theoretical* nutrient and food composition of these diets may help health professionals offering nutrition education and dietary guidance to patients. Many patients may be interested in following some type of plant-based diet or may be open to the suggestion from their healthcare practitioner. Given the more nutrient dense profile of a WFPB diet, patients should be encouraged to pursue such a dietary pattern, with attention given to consuming calcium-rich foods, emphasizing sea vegetables and/or including iodized salt, and using supplements for vitamins B12 and D. In addition, researchers who are interested in dietary adherence can use these estimated targets as a reference standard to determine whether WFPB or vegan diet followers consistently adhere to the dietary advice derived from these sources of cooking and nutrition information. Future research should compare the actual, self-reported intakes of WFPB and vegan followers with these targeted levels to assess adherence. 

## 5. Conclusions

ADAPT study participants who self-identify as either WFPB or vegan have almost identical food and nutrient intake targets, as identified from popular sources of nutrition and cooking information. Based on analyses using NDSR, theoretical dietary intakes of a WFPB diet deviate substantially from MyPlate recommendations. Overall, WFPB diets provide a more nutrient-dense diet than typical US intakes, with less refined grains and added sugars than typical Americans’ diets, though supplements of vitamins B12 and D would be advisable. Future research should examine actual dietary intakes of these groups to assess nutrient composition and adherence to diet recommendations. 

## Figures and Tables

**Table 1:** Comparison of food intakes and diet quality for theoretical whole-food plant-based (WFPB) ^1,2^ meal plans compared to MyPlate ^2^.

	MyPlate		WFPB		% diff. versus MP ^3^
Food Group Servings	Mean	SD	Mean	SD	
Total vegetables (cup)	2.5	0.9	7.0	2.8	+180
Green leafy vegetables (cup)	0.8	0.7	2.7	1.9	+238
Legumes (cup)	0.5	0.6	2.8	1.4	+460
Whole fruits (cup)	1.1	0.6	2.2	1.1	+100
Whole grains (oz)	3.4	1.4	7.9	2.3	+132
Refined grains (oz)	3.4	1.6	0.9	1.2	−74
Nuts and seeds (oz)	0.3	0.5	0.9	0.9	+200
Nut and seed butters (oz)	0.5	0.8	0.4	0.8	−20
Dairy (cups)	3.1	0.5	0.3	0.4	−90
Eggs (oz)	0.7	0.7	0.0	0.0	−100
Poultry (oz)	0.4	1.2	0.0	0.0	−100
Seafood (oz)	1.2	1.6	0.0	0.0	−100
Red meat (oz)	1.2	1.4	0.0	0.0	−100
Meat alternatives (oz)	0.4	1.1	2.9	4.7	+625
**HEI-2015 Score**	100	--	88	--	−12

^1^ Meal plans were generated and theoretical food and nutrient levels were calculated for both diets. Results from WFPB and vegan diets were virtually identical. For those items in which the standard deviation is 50% of the point estimate or more, we examined the median difference with similar findings (data not shown). ^2^ Intakes are standardized to 2000 kcal. ^3^ MP = MyPlate; % difference (greater or lesser) was calculated as % difference = (diet value/MyPlate value) * 100–100.

**Table 2:** Estimated nutrient levels and % differences of WFPB ^1,2^ meal plans compared to MyPlate ^2^.

	MyPlate		WFPB		% diff versus MP ^3^
	Mean	SD	Mean	SD	
**Energy (kcal)**	2000	--	2000	--	--
**Fat (g)**	64	11	38	13	−41
Total fat (% energy)	29	5	17	6	−41
Saturated fat (% energy)	8	2	3	1	−66
MUFA (% energy)	11	2	6	3	−46
PUFA (% energy)	8	2	6	2	−21
Unsat:sat fat ratio	3	1	5	1	+88
**CHO (g)**	272	29	365	34	+34
CHO (% energy)	54	6	73	7	+34
Added sugars (g)	26	15	9	10	−64
Added sugars (% energy)	6	3	2	2	−65
**Protein (g)**	96	11	81	12	−15
Protein (% energy)	19	2	16	2	−15
Animal protein (% energy)	12	2	0	0	−100
Plant protein (% energy)	7	1	16	2	+127
**Fiber** (g)	28	5	70	9	+146
**Micronutrients**					
Dietary vitamin A activity (RAE, µg)	1344	703	1824	1042	+36
Total vitamin D ^4^ (calciferol, µg)	10	5	25	15	+141
Dietary vitamin D ^5^ (calciferol, µg)	10	5	1	1	−91
Dietary vitamin E (Alpha-Tocopherol mg)	15	5	20	5	+35
Total vitamin B124 (µg)	6	3	904	241	+14,349
Dietary vitamin B126 (µg)	6	3	3	5	−54
Dietary folate equivalents (µg)	458	135	935	272	+104
Dietary vitamin C (mg)	134	64	239	152	+78
Dietary calcium (mg)	1434	247	959	273	−33
Dietary magnesium(mg)	419	59	711	75	+70
Dietary potassium (mg)	4071	583	5387	1009	+32
Dietary iron (mg)	15	4	26	4	+79
Dietary sodium (mg)	2301	661	2807	970	+22

^1^ Meal plans were generated and theoretical food and nutrient levels were calculated for both diets. Results from WFPB and vegan diets were virtually identical. For those items in which the standard deviation is 50% of the point estimate or more, we examined the median difference with similar findings (data not shown). ^2^ Standardized to 2000 kcal. ^3^ Percent difference was calculated as % difference = (diet value/recommended value)*100 − 100. ^4^ Total vitamin D and B12 includes that from foods (including fortified foods) and supplements. Dietary vitamin D and B12 includes only that from foods (including fortified foods). ^5^ Median, quartile 1, and quartile 2 of dietary vitamin D were as follows: 0 µg, 0 µg, 2 µg. Means are presented in the table to reflect adjustments made in absorption based on status. ^6^ Median, quartile 1, and quartile 2 of dietary vitamin B12 were as follows: 1 µg, 0 µg, 2 µg. Means are presented in the table to reflect adjustments made in absorption based on status.

**Table 3:** Estimated levels of nutrients of public health concern and % differences (greater or lesser) of WFPB diets compared to recommendations.^1, 6.^

	Men						Women					
	RDA	Diff	EAR	Diff	AI	Diff	RDA	Diff	EAR	Diff	AI	Diff
Vitamin A (μg)	900	+95	625	+192	-	-	700	+151	500	+265	-	-
Vitamin D ^2^ (μg)	15	+80	10	+150	-	-	15	+80	10	+150	-	-
Vitamin E (mg)	15	+33	12	+67	-	-	15	+33	12	+67	-	-
Folate total (μg)	400	+134	320	+192	-	-	400	+134	320	+192	-	-
Vitamin C (mg)	90	+177	75	+219	-	-	75	+232	60	+298	-	-
Calcium ^3^ (mg)	1000	−2 *	800	+20	-	-	1000–1200	−18 *	800–1000	−4 *	-	-
Magnesium ^4^ (mg)	400–420	+69	330–350	+103	-	-	310–320	+122	255–265	+168	-	-
Iron ^5^ (mg)	8	+225	6	+333	-	-	8–18	+44	8.1–5	+221	-	-
Potassium (g)	-	-	-	-	4.7	+15	-	-	-	-	4.7	+15
Fiber ^7^ (g)	-	-	-	-	30–38	+84	-	-	-	-	21–25	+180
Sodium (g)	-	-	-	-	2.3	+22 *	-	-	-	-	2.3	+22 *
	Dietary Guidelines for Americans (men and women)				-	-	-	-	-	-	-	-
Saturated fat (% kcal)	<10%	-	−70	-	-							
Added sugar (% kcal)	<10%	-	−80	-								

^1^ Recommended levels applied to all men and women except where noted. The highest recommended level for a subgroup was used for comparison. % difference was calculated as: % difference = (meal plan value/recommended value) * 100 − 100. ^2^ Total vitamin D (both food and supplements). ^3^ Comparison applies to all men and women ages 51–70 years (RDA 1200 mg/day; EAR 1000 mg/day). ^4^ Comparison applies to all men ages 31–70 years (RDA 420 mg/day; EAR 350 mg/day) and women ages 31–70 years (Recommended Dietary Allowance (RDA) 320 mg/day; 265 mg/day). ^5^ Comparison applies to all men (RDA 8 mg/day; EAR 6 mg/day) and women ages 19–50 years (RDA 18 mg/day; EAR 8.1 mg/day). ^6^ Estimated Average Requirement (EAR) for vitamins A, D, E, C, folate, calcium, magnesium, iron; Adequate Intake (AI) for potassium and fiber; Upper Limit (UL) for sodium; and Dietary Guidelines for Americans recommended limits for saturated fat and added sugar. ^7^ Comparison applies to men and women ages 19–50 years (men: 38 g/day; women: 25 g/day). * Does not meet recommendations.

## Supplementary Materials

The following are available online at https://www.mdpi.com/2072-6643/11/3/625/s1: Table S1: HEI–20151 Components & Scoring Standards; Table S2: Comparison of Food Intakes and Diet Quality for Theoretical Vegan1,2 Meal Plans Compared to MyPlate; Table S3: Estimated Nutrient Levels and % Differences of Vegan1,2 Meal Plans Compared to MyPlate; Table S4: Estimated Levels of Nutrients of Public Health Concern and % Differences (Greater or Lesser) of Vegan Diets Compared to Recommendations; Table S5: Medians and Interquartile Ranges for Mean Nutrient Intakes from MyPlate, WFPB, and Vegan Meal Plans.
