# Supplementary material for: Theoretical Food and Nutrient Composition of Whole-Food Plant-Based and Vegan Diets Compared to Current Dietary Recommendations
Source: Nutrients. 2019 Mar 14;11(3):625. doi: 10.3390/nu11030625 (PMC6471973; doi:10.3390/nu11030625)
Supplement: Supplementary file 1 [file nutrients-11-00625-s001.pdf]

Article

Theoretical Food and Nutrient Composition of Whole-Food Plant Based and Vegan Diets Compared to Current Dietary Recommendations

Supplementary Materials:

Table S1. HEI–2015[1] Components & Scoring Standards
(https://epi.grants.cancer.gov/hei/developing.html).

Component	Maximum	Standard for maximum	Standard for minimum score of
**Adequacy:**			
**Total Fruits[2]**	5	≥0.8 cup equiv. per 1,000	No Fruit
**Whole Fruits[3]**	5	≥0.4 cup equiv. per 1,000	No Whole Fruit
**Total Vegetables[4]**	5	≥1.1 cup equiv. per 1,000	No Vegetables
**Greens and Beans[4]**	5	≥0.2 cup equiv. per 1,000	No Dark Green Vegetables or
**Whole Grains**	10	≥1.5 oz equiv. per 1,000	No Whole Grains
**Dairy[5]**	10	≥1.3 cup equiv. per 1,000	No Dairy
**Total Protein Foods[6]**	5	≥2.5 oz equiv. per 1,000	No Protein Foods
**Seafood and Plant**	5	≥0.8 oz equiv. per 1,000	No Seafood or Plant Proteins
**Fatty Acids[8]**	10	(PUFAs + MUFAs)/SFAs	(PUFAs + MUFAs)/SFAs ≤1.2
**Moderation:**			
**Refined Grains**	10	≤1.8 oz equiv. per 1,000	≥4.3 oz equiv. per 1,000 kcal
**Sodium**	10	≤1.1 gram per 1,000 kcal	≥2.0 grams per 1,000 kcal
**Added Sugars**	10	≤6.5% of energy	≥26% of energy
**Saturated Fats**	10	≤8% of energy	≥16% of energy

[1] Intakes between the minimum and maximum standards are scored proportionately.
[2] Includes 100% fruit juice.
[3] Includes all forms except juice.
[4] Includes legumes (beans and peas).
[5] Includes all milk products, such as fluid milk, yogurt, and cheese, and fortified soy beverages.
[6] Includes legumes (beans and peas).
[7] Includes seafood, nuts, seeds, soy products (other than beverages), and legumes (beans and peas).
[8] Ratio of poly- and monounsaturated fatty acids (PUFAs and MUFAs) to saturated fatty acids (SFAs).

Table S2. Comparison of Food Intakes and Diet Quality for Theoretical Vegan[1,2] Meal Plans Compared to MyPlate[2.]

	**MyPlate**		**Vegan**		**% diff. vs. MP[3]**
**Food Group Servings**	**Mean**	**SD**	**Mean**	**SD**	

Total vegetables (cup)	2.5	0.9	7.1	2.8	+184
Green leafy vegetables (cup)	0.8	0.7	2.8	1.9	+250
Legumes (cup)	0.5	0.6	2.9	1.4	+480
Whole fruits (cup)	1.1	0.6	2.2	1.0	+100
Whole grains (oz)	3.4	1.4	7.9	2.4	+132
Refined grains (oz)	3.4	1.6	0.9	1.3	-74
Nuts and seeds (oz)	0.3	0.5	1.0	1.0	+233
Nut and seed butters (oz)	0.5	0.8	0.4	0.8	-20
Dairy (cups)	3.1	0.5	0.3	0.4	-90
Eggs (oz)	0.7	0.7	0.0	0.0	-100
Poultry (oz)	0.4	1.2	0.0	0.0	-100
Seafood (oz)	1.2	1.6	0.0	0.0	-100
Red meat (oz)	1.2	1.4	0.0	0.0	-100
Meat alternatives (oz)	0.4	1.1	2.5	3.8	+525
**HEI-2015 Score**	100	--	88	--	-12

[1] Meal plans were generated and theoretical food and nutrient levels were calculated for both diets. Results from WFPB and vegan diets were virtually identical. For those items in which the standard deviation is 50% of the point estimate or more, we examined the median difference with similar findings (data not shown).
[2] Intakes are standardized to 2000 kcal
[3] MP=MyPlate; % difference (greater or lesser) was calculated as % difference = (diet value / MyPlate value)*100 – 100.

Table S3. Estimated Nutrient Levels and % Differences of Vegan[1,2] Meal Plans Compared to MyPlate[2].

	**MyPlate**		**Vegan**		**% diff vs. MP[3]**
	**Mean**	**SD**	**Mean**	**SD**	
**Energy (kcal)**	2000	--	2000	--	--
**Fat (g)**	64	11	39	13	-39
Total fat (% energy)	29	5	17	6	-39
Saturated fat (% energy)	8	2	3	1	-66
MUFA (% energy)	11	2	6	3	-44
PUFA (% energy)	8	2	7	2	-19
Unsat:sat fat ratio	3	1	5	1	+92
**CHO (g)**	272	29	363	34	+33
CHO (% energy)	54	6	73	7	+33
Added sugars (g)	26	15	9	9	-66
Added sugars (% energy)	6	3	2	2	-66
**Protein (g)**	96	11	81	11	-16
Protein (% energy)	19	2	16	2	-16

Animal protein (% energy)	12	2	0	0	-100
Plant protein (% energy)	7	1	16	2	+126
**Fiber** (g)	28	5	70	8	+147
**Micronutrients**					
Dietary Vitamin A Activity (RAE, μg)	1344	703	1859	1073	+38
Total Vitamin D[4] (calciferol, μg)	10	5	27	14	+169
Dietary Vitamin D[5] (calciferol, μg)	10	5	1	1	-91
Dietary Vitamin E (Alpha-Tocopherol mg)	15	5	20	5	+35
Total Vitamin B12[4] (μg)	6	3	880	265	+13964
Dietary Vitamin B12[6] (μg)	6	3	2	4	-61
Dietary folate equivalents (μg)	458	135	965	275	+110
Dietary vitamin C (mg)	134	64	244	147	+82
Dietary calcium (mg)	1434	247	962	263	-33
Dietary magnesium(mg)	419	59	716	77	+71
Dietary potassium (mg)	4071	583	5402	1045	+33
Dietary iron (mg)	15	4	26	4	+81
Dietary sodium (mg)	2301	661	2743	899	+19

[1] Meal plans were generated and theoretical food and nutrient levels were calculated for both diets. Results from WFPB and vegan diets were virtually identical. For those items in which the standard deviation is 50% of the point estimate or more, we examined the median difference with similar findings (data not shown).

[2] Standardized to 2000 kcal.

[3] Percent difference was calculated as % difference = (diet value / recommended value)*100 – 100.

[4] Total vitamin D and B12 includes that from foods (including fortified foods) and supplements. Dietary vitamin D and B12 includes only that from foods (including fortified foods).

[5] Median, quartile 1, and quartile 2 of dietary vitamin D were as follows: 0μg, 0μg, 2μg. Means are presented in the table to reflect adjustments made in absorption based on status.

[6] Median, quartile 1, and quartile 2 of dietary vitamin B12 were as follows: 1μg, 0μg, 2μg. Means are presented in the table to reflect adjustments made in absorption based on status.

Table S4. Estimated Levels of Nutrients of Public Health Concern and % Differences (Greater or Lesser) of Vegan Diets Compared to Recommendations.

	Men						Women					
	RDA	Diff	EAR	Diff	AI	Diff	RDA	Diff	EAR	Diff	AI	Diff
Vitamin A (μg)	900	+107	625	+197			700	+166	500	+272		
Vitamin D[2] (μg)	15	+80	10	+170			15	+80	10	+170		
Vitamin E (mg)	15	+33	12	+67			15	+33	12	+67		
Folate total (μg)	400	+141	320	+202			400	+141	320	+202		
Vitamin C (mg)	90	+171	75	+225			75	+225	60	+307		
Calcium[3] (mg)	1000	-4*	800	+20			1000-1200	-20*	800-1000	-4*		
Magnesium[4] (mg)	400-420	+70	330-350	+105			310-320	+124	255-265	+170		
Iron[5] (mg)	8	+225	6	+333			8-18	+44	8.1-5	+221		
Potassium (g)					4.7	+15					4.7	+15
Fiber[7] (g)					30-38	+84					21-25	+180
Sodium (g)					2.3	+19*					2.3	+19*

	Dietary Guidelines for Americans (men and women)		
Saturated fat (% kcal)	<10%	-70	
Added sugar (% kcal)	<10%	-80	

[1] Recommended levels applied to all men and women except where noted. The highest recommended level for a subgroup was used for comparison. % difference was calculated as: % difference = (meal plan value / recommended value) * 100 – 100.

[2] Total vitamin D (both food and supplements)

[3] Comparison applies to all men and women ages 51-70y (RDA 1,200 mg/d; EAR 1,000 mg/d)

[4] Comparison applies to all men ages 31-70y (RDA 420 mg/d; EAR 350 mg/d) and women ages 31-70y (RDA 320 mg/d; 265 mg/d)

[5] Comparison applies to all men (RDA 8mg/d; EAR 6 mg/d) and women ages 19-50y (RDA 18mg/d; EAR 8.1 mg/d)

[6] Estimated Average Requirement (EAR) for vitamins A, D, E, C, folate, calcium, magnesium, iron; Adequate Intake (AI) for potassium and fiber; Upper Limit (UL) for sodium; and Dietary Guidelines for Americans recommended limits for saturated fat and added sugar

[7] Comparison applies to men and women ages 19-50y (men: 38 g/d; women: 25g/d)

* Does not meet recommendations

Table S5. Medians and Interquartile Ranges for Mean Nutrient Intakes from MyPlate, WFPB, and Vegan Meal Plans[1,2].

	MyPlate			WFPB			Vegan		
	Medi	Q1	Q3	Media	Q1	Q3	Media	Q1	Q3
**Food Group Servings**									
Total vegetables (cup)	2.5	1.8	3.3	6.3	5.3	9.1	6.3	5.3	8.7
Green leafy vegetables	0.8	0.0	1.2	2.5	1.2	3.2	2.5	1.5	3.3
Legumes (cup)	0.4	0.0	0.8	2.9	1.7	3.5	3.1	1.7	4.0
Whole fruits (cup)	0.9	0.7	1.5	2.1	1.4	2.6	2.1	1.4	2.6
Whole grains (oz)	3.1	2.6	3.8	7.1	6.1	10.0	7.1	6.1	10.7
Refined grains (oz)	3.5	2.3	4.4	0.2	0.0	1.3	0.2	0.0	0.9
Nuts and seeds (oz)	0.0	0.0	0.3	0.7	0.3	1.0	0.7	0.3	1.5
Nut and seed butters (oz)	0.0	0.0	0.9	0.0	0.0	0.4	0.0	0.0	0.4
Dairy (cups)	3.1	2.9	3.4	0.1	0.0	0.8	0.1	0.0	0.5
Eggs (oz)	0.5	0.0	1.1	0.0	0.0	0.0	0.0	0.0	0.0
Poultry (oz)	0.0	0.0	0.0	0.0	0.0	0.0	0.0	0.0	0.0
Seafood (oz)	0.0	0.0	2.6	0.0	0.0	0.0	0.0	0.0	0.0
Red meat (oz)	0.9	0.0	2.5	0.0	0.0	0.0	0.0	0.0	0.0
Meat alternatives (oz)	0.0	0.0	0.0	1.3	0.0	4.0	1.8	0.0	4.0
**Nutrients**	**Medi**	**Q1**	**Q3**	**Media**	**Q1**	**Q3**	**Media**	**Q1**	**Q3**
**Energy (kcal)**	--	--	--	--	--	--	--	--	--
**Fat (g)**	64	57	70	35	31	43	35	31	42
Total fat (% energy)	29	26	31	16	14	19	16	14	19
Saturated fat (% energy)	8	6	9	2	2	3	2	2	3
MUFA (% energy)	10	10	12	5	3	8	5	4	8
PUFA[3] (% energy)	8	6	10	6	5	7	6	5	7
Ratio unsat:sat fat	2	2	3	5	4	6	5	4	6
**CHO (g)**	272	257	289	374	361	386	373	361	380
CHO (% energy)	54	51	58	75	72	77	75	72	76
Added sugars (g)	26	15	31	5	1	17	5	1	13
Added sugars (% energy)	6	4	8	1	0	4	1	0	3
**Protein (g)**	96	89	102	81	76	86	81	75	85
Protein (% energy)	19	18	20	16	15	17	16	15	17
Animal protein (%	11	10	13	0	0	0	0	0	0
Plant protein (% energy)	7	6	8	16	15	17	16	15	17
**Fiber** (g)	28	26	31	69	63	76	69	63	76
**Micronutrients**									
Total Vitamin A Activity	1216	820	1700	1612	996	2285	1680	996	2164
Total Vitamin D[3]	9	7	10	25	24	29	25	25	29
Dietary Vitamin D	9	7	10	0	0	2	0	0	2
Dietary Vitamin E	14	12	16	21	15	22	21	15	22
Total Vitamin B12[3] (μg)	6	5	6	994	979	999	993	962	999
Dietary Vitamin B12	6	5	6	1	0	2	1	0	2
Dietary folate equivalents	464	355	512	1002	730	1143	1005	730	1143
Dietary vitamin C (mg)	114	87	187	211	149	274	211	149	353
Dietary calcium (mg)	1428	127	1613	999	719	1075	999	774	1084
Total Magnesium[2] (mg)	395	376	444	709	657	780	710	659	803
Dietary	395	376	444	709	657	776	709	659	780
Dietary Potassium (mg)	4056	361	4374	5131	4716	5678	5131	4716	5661
Dietary Iron (mg)	14	12	18	26	22	30	26	24	30
Dietary Sodium (mg)	2205	187	2852	2731	2069	3131	2731	2069	3108

[1] Meal plans were generated and theoretical food and nutrient levels were calculated for both diets.

[2] Intakes are standardized to 2000 kcal

[3] Total vitamin D and B12 includes that from foods (including fortified foods) and supplements. Dietary vitamin D and B12 includes only that from foods (including fortified foods).
